# Primary health care, the Declaration of Astana and COVID-19

**DOI:** 10.2471/BLT.20.252932

**Published:** 2020-10-05

**Authors:** Kumanan Rasanathan, Tim G Evans

**Affiliations:** a Health Systems Global, 36 St 310, Phnom Penh, Cambodia.; b School of Population and Global Health, McGill University, Montreal, Canada.

## Abstract

Four decades after the Declaration of Alma-Ata, its vision of health for all and strategy of primary health care are still an inspiration to many people. In this article we evaluate the current status of primary health care in the era of the Declaration of Astana, the sustainable development goals, universal health coverage and the coronavirus disease 2019 pandemic. We consider how best to guide greater application of the primary health care strategy, reflecting on tensions that remain between the political vision of primary health care and its implementation in countries. We also consider what is required to support countries to realize the aspirations of primary health care, arguing that national needs and action must dominate over global preoccupations. Changing contexts and realities need to be accommodated. A clear distinction is needed between primary health care as an inspirational vision and set of values for health development, and primary health care as policy and implementation space. To achieve this vision, political action is required. Stakeholders beyond the health sector will often need to lead, which is challenging because the concept of primary health care is poorly understood by other sectors. Efforts on primary health care as policy and implementation space might focus explicitly on primary care and the frontline of service delivery with clear links and support to complementary work on social determinants and building healthy societies. Such efforts can be partial but important implementation solutions to contribute to the much bigger political vision of primary health care.

## Résumé

Quarante ans après la Déclaration d'Alma-Ata, sa vision en matière de santé universelle et sa stratégie de soins de santé primaires demeurent une inspiration pour de nombreuses personnes. Dans cet article, nous évaluons l'état actuel des soins de santé primaires à l'ère de la Déclaration d'Astana, des objectifs de développement durable, de la couverture maladie universelle et de la pandémie de maladie à coronavirus 2019 (COVID-19). Nous tentons de déterminer quel est le meilleur moyen de favoriser une application plus vaste de la stratégie de soins de santé primaires, en tenant compte des tensions qui subsistent entre la vision politique des soins de santé primaires et leur mise en œuvre dans les différents pays. Nous identifions également les éléments qui aident les pays à concrétiser les aspirations liées aux soins de santé primaires, et affirmons que les besoins et actions à l'échelle nationale doivent primer sur les préoccupations internationales. L'évolution des contextes et des réalités doit être prise en considération. Il est impératif d'opérer une nette distinction entre les soins de santé primaires comme source d'inspiration et ensemble de valeurs guidant le développement sanitaire d'une part, et comme espace politique et de mise en œuvre de l'autre. Pour y parvenir, des actes politiques sont indispensables. Des intervenants n'appartenant pas au secteur de la santé devront souvent prendre l'initiative, ce qui représente un défi car le concept des soins de santé primaires suscite l'incompréhension dans les autres secteurs. Les efforts en matière d'espace politique et de mise en œuvre pourraient se concentrer explicitement sur les soins de santé primaires et la première ligne des prestations de service, avec des liens clairement établis et un soutien aux travaux complémentaires consacrés aux déterminants sociaux et à la création d'une société saine. De tels efforts peuvent offrir des solutions partielles mais essentielles à l'élaboration d'une vision politique bien plus large des soins de santé primaires.

## Resumen

Cuatro décadas después de la Declaración de Almá Atá, su visión de la salud para todos y su estrategia de atención primaria de salud siguen siendo una inspiración para muchas personas. En este artículo se evalúa el estado actual de la atención primaria de salud en la era de la Declaración de Astaná, los objetivos de desarrollo sostenible, la cobertura sanitaria universal y la pandemia de la enfermedad por coronavirus de 2019. Se analiza la mejor manera de orientar una mayor aplicación de la estrategia de atención primaria de salud al estudiar las tensiones que subsisten entre la visión política de la atención primaria de salud y su aplicación en los países. También se analiza lo que se requiere para ayudar a los países a materializar las aspiraciones de la atención primaria de salud al argumentar que las necesidades y las medidas nacionales deben prevalecer sobre las preocupaciones mundiales. Se deben tener en cuenta los contextos y las realidades cambiantes. Hay que establecer una clara diferencia entre la atención primaria de salud como visión inspiradora y conjunto de valores para el desarrollo de la salud, y la atención primaria de salud como entorno normativo y de aplicación. Por consiguiente, se requiere la adopción de medidas políticas para hacer realidad esta visión. Con frecuencia, las partes interesadas que no pertenecen al sector sanitario tendrán que tomar la iniciativa, lo que supone un reto porque el concepto de atención primaria de salud no se comprende bien en otros sectores. Los esfuerzos relacionados con la atención primaria como entorno normativo y de aplicación se podrían centrar de manera explícita en la atención primaria y en la prestación de servicios de primera línea a través de vínculos claros y el apoyo a la labor complementaria sobre los determinantes sociales y la construcción de sociedades sanas. Esos esfuerzos pueden ser soluciones parciales pero importantes de aplicación para contribuir a la visión política mucho más amplia de la atención primaria de salud.

## ملخص

بعد أربعة عقود من إعلان ألما آتا (Declaration of Alma-Ata)، لا تزال رؤيته الصحية، واستراتيجيته للرعاية الصحية الأولية، مصدرين للإلهام للعديد من الناس. نقوم في هذا المقال بتقييم الوضع الحالي للرعاية الصحية الأولية في حقبة إعلان أستانا، وأهداف التنمية المستدامة، والتغطية الصحية الشاملة، وجائحة فيروس كورونا 2019. نحن نفكر في أفضل وسيلة نحو تطبيق أكبر لاستراتيجية الرعاية الصحية الأولية، مع التأمل في التوترات المتبقية بين الرؤية السياسية للرعاية الصحية الأولية، وتنفيذها في البلدان. كما نفكر أيضًا فيما هو مطلوب لدعم البلدان لتحقيق تطلعات الرعاية الصحية الأولية، مع الدفع بأن الاحتياجات والإجراءات الوطنية يجب تغليبها على الشواغل العالمية. كما يجب مراعاة السياقات والحقائق المتغيرة. هناك حاجة إلى تمييز واضح بين الرعاية الصحية الأولية باعتبارها رؤية ملهمة ومجموعة من القيم للتنمية الصحية، والرعاية الصحية الأولية، كمساحة للسياسة والتنفيذ. لتحقيق هذه الرؤية، هناك حاجة للعمل السياسي. غالبًا ما يحتاج أصحاب المصلحة خارج قطاع الصحة إلى القيادة، وهو ما يمثل تحديًا لأن مفهوم الرعاية الصحية الأولية غير مفهوم جيدًا لدى القطاعات الأخرى. قد تركز الجهود المبذولة في مجال الرعاية الصحية الأولية كمساحة للسياسة والتنفيذ بشكل صريح على الرعاية الأولية، والخط الأمامي لتقديم الخدمات، مع روابط واضحة ودعم للعمل التكميلي على المحددات الاجتماعية وبناء مجتمعات صحية. يمكن أن تكون هذه الجهود جزئية، ولكنها حلول تنفيذ هامة للمساهمة في رؤية سياسية أوسع بكثير للرعاية الصحية الأولية.

## 摘要

在《阿拉木图宣言》提出四十年之后，其关于人人享有健康的愿景和初级卫生保健策略，现在仍然激励着许多人。本文中，我们评估了《阿斯塔纳宣言》时代的初级卫生保健现状、可持续发展目标、全民健康保险和 2019 年新型冠状病毒肺炎的现状。我们考虑如何最好地指导更广泛地实施初级卫生保健策略，这反应了初级卫生保健的政治愿景与各国对这一战略的实施状况之间仍存在的冲突。我们还考虑了支持各国实现初级卫生保健愿景所需的条件，认为国家需要和行动必须在全球预防措施中占主导地位。需适应不断变化的环境和现实情况。需明确区分初级卫生保健作为鼓励性愿景和卫生发展价值观体系，以及初级卫生保健作为政策和实施空间之间的差别。实现该愿景需采取政治措施。卫生部门外利益相关方经常需要带头，这具有一定挑战性，因为其它部门缺乏对初级卫生保健概念的了解。初级卫生保健作为政策和实施空间方面的工作，必须重点关注初级护理和服务工作一线，为辅助确定社会决定因素和构建健康社会工作提供明确的联系和支持。此项工作是实施解决方案的一部分，但是非常重要，有助于实现更大的初级卫生保健政治愿景。

## Резюме

Спустя сорок лет после принятия Алма-Атинской декларации изложенные в ней концепция «здоровье для всех» и стратегия предоставления первичной медико-санитарной помощи по-прежнему служат источником вдохновения для многих людей. В этой статье авторы оценивают текущее состояние системы первичной медико-санитарной помощи в эпоху Астанинской декларации, цели устойчивого развития, всеобщий охват услугами здравоохранения и пандемию коронавирусного заболевания 2019 года. Авторы рассматривают, как наилучшим образом обеспечить более широкое применение стратегии первичной медико-санитарной помощи, и обсуждают сохраняющиеся противоречия между политическим видением системы первичной медико-санитарной помощи и ее реализацией в странах. Авторы также рассматривают вопрос о том, какая поддержка необходима странам в реализации целей в области первичной медико-санитарной помощи, утверждая, что национальные потребности и действия должны превалировать над озабоченностью глобальными проблемами. Нужно научиться приспосабливаться к изменяющимся условиям и реалиям. Необходимо четко разграничить первичную медико-санитарную помощь как вдохновляющую концепцию и набор ценностей для развития здравоохранения и первичную медико-санитарную помощь как площадку для разработки и реализации политики. Для достижения этой цели необходимы политические действия. Ведущая роль часто достается заинтересованным сторонам, не имеющим отношения к сектору здравоохранения, что осложняет задачу, поскольку представители других секторов не имеют правильного представления о концепции первичной медико-санитарной помощи. Усилия в области обеспечения первичной медико-санитарной помощи как площадки для разработки и реализации политики могут быть сосредоточены непосредственно на первичной медико-санитарной помощи и первой линии оказания услуг с определением четких связей и поддержкой дополнительной работы по социальным детерминантам и построению здорового общества. Такие усилия могут быть частичными, но важными решениями по реализации целей, способствующих более широкому политическому видению концепции первичной медико-санитарной помощи.

## Introduction

In 2018, the global health community came together in Astana, Kazakhstan to celebrate the 40th anniversary of the 1978 International Conference on Primary Health Care and the ensuing Declaration of Alma-Ata.^1^ The Declaration has influenced a generation of public health workers towards health for all through the strategy of primary health care. Among the primary tenets of the Declaration were: health as a human right; communities driving decisions that influence their health; health care close to where people live; and coordinated efforts across society to create health, including fairer social and economic arrangements. The Declaration also emphasized the importance of primary care services. Primary care serves as a person’s first point of contact when people seek health care, engaging their family and community context, dealing with most problems, and acting as the fulcrum of the health system, referring patients onward to other services when necessary.^2^ None of these principles have lost their relevance over time.^3^ The Alma-Ata Declaration’s influence and canonical status in public health are unmatched by almost any other text, and certainly in comparison with the many declarations, statements and resolutions issued each year by the global health community.

Every 10 years the World Health Organization (WHO), with the support of the United Nations Children’s Fund, has led efforts to celebrate the Alma-Ata Declaration and reinvigorate the primary health care movement. For the 40th anniversary in 2018, the Declaration of Astana, along with a range of background documents and analyses, aimed to reassert the relevance of primary health care while also updating the Alma-Ata vision.^4^^–^^11^ A similar effort was undertaken in 2008 for the 30th anniversary of the Alma-Ata Declaration.^12^^–^^16^

Despite these regular efforts to rejuvenate the Alma-Ata vision, no single country, let alone the whole world, has achieved the target of health for all by the year 2000 as defined at Alma-Ata.^17^ Many countries have successfully implemented the directions advocated in the Declaration. Mostly these are high-income countries but there are also key success stories in low- and middle-income countries.^18^ Yet countervailing forces^12^ have led in many instances to the marginalization of primary health care. This marginalization has resulted in insufficient prioritization of primary care services, inattention to the impact of other sectors on health, and lack of progress on health equity.^19^ For example, in the current pandemic of coronavirus disease 2019 (COVID-19), many countries have overlooked primary care services and the primary health-care strategy as the vehicle for tackling the disease outbreak.

Here we aim to evaluate the current status of primary health care as a vehicle towards health for all in the era of the Astana Declaration, the sustainable development goals (SDGs),^20^ universal health coverage (UHC)^20^ and COVID-19.^21^ We propose that the Alma-Ata Declaration may be seen as the palimpsest of global health. A palimpsest is a manuscript, papyrus or other form of writing material which has been used repeatedly with the latest writing superimposed on earlier attempts. At each anniversary of the Alma-Ata Declaration, the global health community has reinterpreted and rewritten the concept of primary health care, in an attempt to address the lack of progress towards the vision of health for all. Earlier versions have been only partially erased. The palimpsest therefore bears visible traces of its earlier text underneath the latest version – a record of the repeated efforts of expression. We consider how the most recent interpretation of primary health care at the Astana conference can best guide greater application of the primary health-care strategy. We examine tensions, remaining since the Alma-Ata conference, between the broad political vision of primary health care and its implementation in countries, which has mostly focused on primary care. We also consider what support countries need to apply primary health care more effectively to accomplish health for all.

## Changes in health since Alma-Ata

The context for health has changed considerably since 1978, sometimes in ways not anticipated at the Alma-Ata conference. Due in part to successes of the primary health-care strategy, maternal and child mortality globally have fallen dramatically,^17^ with a reduced relative burden of disease from communicable diseases. However, the noncommunicable disease burden (not mentioned in the Alma-Ata Declaration) has increased, now accounting for an estimated 41 million deaths each year globally, over 70% of the global disease mortality burden, and therefore poses a much greater need for delivery of chronic care services.^22^

The changing disease burden over time itself reflects multiple other transitions that have occurred or are occurring at different speeds within countries. Factors that impose new and often greater demands on health systems since 1978 include: economic growth (so that aid has become a relatively smaller proportion of the health budget in most countries); widening inequality in some countries; falling fertility rates and the resulting ageing populations; polarization of societies and persisting conflicts; increasing mobility of populations; and the health impacts of climate change and environmental degradation. These changes require complex and coordinated multisectoral action which must overcome powerful political and commercial interests.^19^

The information technology revolution has transformed societies and individuals, including the health sector, in the 40 years since the Alma-Ata conference. These changes provide major opportunities to transform care, especially primary care, with better tracking of people’s health and new channels for service delivery including telemedicine. Mobile phones and the internet make information directly accessible to people, including poor and remote populations who have never had access to phones or computers before. But at the same time, these trends bring potential challenges. The greater availability of misinformation and awareness of inequity and what some people receive can increase people’s expectations, dissatisfaction and distrust with health services. The increasing use of these technologies in the health sector can also exacerbate health inequities, due to inequities in access to information and communications technologies. The potential and threats of artificial intelligence, machine learning and the systematic extraction and analysis of large and complex data remain to be realized and understood.

Health sectors in most countries are currently pluralistic. The for-profit and not-for-profit sectors play a greater role in health service delivery and the production of health commodities – and have greater influence on the determinants of health^23^ – than was originally recognized by the Alma-Ata Declaration. In many countries, the private sector dominates health service delivery with varying degrees of effectiveness, quality and efficiency of care. There are often negative impacts on equity. Government stewardship and regulation of the private sector is weak in most countries.^24^

The 40 years since the Alma-Ata conference have seen an acceleration of the emergence of new infectious diseases, highlighted by the current COVID-19 pandemic. Yet the Alma-Ata Declaration was relatively silent on what is now described as health security, and indeed on population-level health services (sometimes described as essential public health functions). There is now greater understanding of the value of integrating primary care and public health services, and of the importance of strengthening primary care and the role of communities in emergency surveillance, preparedness and response.

## Primary health care at Astana

In attempting to respond to this changed context, the Astana Declaration recommitted to the principles of the Alma-Ata Declaration.^4^ In the accompanying vision document, three components of primary health care were described: (i) primary care and essential public health functions as the core of integrated health services; (ii) empowered people and communities; and (iii) multisectoral policy and action.^5^

The first component focused on what has been the main policy and implementation space for primary health care since Alma-Ata: primary care, ideally delivered as close as possible to where people live through a people-centred approach.^3^^,^^25^ The Astana Declaration also highlighted the need for essential public health functions to be seen as an integral part of primary health care. These functions include health promotion and surveillance, and preparedness and response for health emergencies including outbreaks.

The second component of primary health care focused on the right of people to be autonomous and in control of their own health, and on efforts to address people’s health needs. People need support to increase their health literacy to protect and promote their own health, through their own choices and also through policies that positively impact on the determinants of health. People should also be able to participate in the way health services are managed and delivered. This involvement includes being able to raise concerns which are acted upon, and to participate in decisions about tailoring health services to the needs of specific communities. People belong to multiple communities that can serve as nodes for action and solidarity in both the governance of health services and service delivery itself, including through community health workers (CHWs).

The third component – multisectoral policy and action – built upon and expanded the Alma-Ata Declaration concept of intersectoral action, and reflects the greater understanding of the importance of the determinants of health for improving health outcomes and health equity.^26^ The modern concept of multisectoral action as part of primary health care recognizes the important roles of sectors beyond health in creating and destroying health, and the need for coordinated action across these sectors to achieve health goals and reduce health threats, as described in the health in all policies approach.^27^

In these ways, the Astana Declaration aims to clarify the equivocation in the Alma-Ata Declaration between the political vision of primary health care (realizing the right to health to achieve health for all) and the implementation focus of primary health care (comprising primary care and community involvement). The political vision of primary health care is celebrated as still relevant and even essential to achieve the newer constructs of UHC and the SDGs. While most of the implementation of primary health care has been through primary care, there is still a belief that primary health care can be a broader vehicle for instituting actions across society to achieve the SDGs.^8^^,^^10^

## Is primary health care still relevant? 

There have been many overviews of the distinction between primary health care and primary care.^2^^,^^28^^,^^29^ There remains, however, a demand for a more specific definition of primary health care in the context of a specific country. The breadth and complexity of the big picture of health for all, and the ambiguities in the Alma-Ata Declaration, have led many countries to embrace a narrower scope of primary health-care implementation. This narrowing is reflected by the use of terms such as primary health-care workers, primary health-care systems and primary health-care services. There are now newer versions of the trend to prescribe a list of priority interventions for primary health care in terms of primary care and public health interventions.^9^ Such efforts allude to the concept of selective primary health care,^30^^,^^31^ for example in the use of terms such as essential UHC to describe priority packages. It should be noted that selective primary health care was also first articulated as an interim strategy towards the full vision of primary health care. Delimiting primary health care in this way may appear to be more within reach and of immediate relevance to resource-constrained health ministries. But doing so risks missing the opportunity of repositioning health as a whole-of-society endeavour that is necessary for social justice and economic and environmental sustainability.

There remains a debate on the extent to which primary health care encompasses multisectoral action on the determinants of health.^9^^,^^29^ Primary health care may not be sufficiently robust or comprehensible to those beyond the health sector to enable the range of multisectoral actions for health required to achieve goals such as the SDGs related to health. Primary health care as a term sometimes remains unclear to many people within the health sector – it is incomprehensible to most of those beyond the health sector whose leadership is essential to address the determinants of health.

Do these tensions and apparently divergent views between the political vision and the focus on implementation of primary health care matter? For Halfdan Mahler, Director-General of WHO at the time of the Alma-Ata conference, there was no tension: health for all was the symphony and primary health care the score. Yet these ambiguities matter for countries trying to implement the primary health-care strategy; they also explain the partial focus of many primary health-care efforts. A useful contribution of the celebrations of the Alma-Ata Declaration every 10 years has been to consider how to adapt the vision and strategies of primary health care to changing political, social and economic contexts and how to negotiate the politics of health of each new era.

A regular recommitment to the values and political vision of primary health care is important in itself. Nevertheless, there is more to do to identify and define what should be different about the quest for health for all in 2020 compared with 2008, let alone 1978, particularly in terms of what is required to support country efforts. The slowness in adopting primary health care to tackle COVID-19 in many countries crystallizes this challenge.

## Priorities for support to countries

After the Astana celebration there was enthusiasm that “efforts to reinvigorate primary health care are more likely to succeed than in the past.”^7^ We agree that there are grounds for optimism, but also note with caution that similar hopes were held at previous anniversaries (as we know from our own experience of the 2008 attempt at renewal of primary health care). The aspirations towards equity and comprehensiveness of care encapsulated in primary health care face many political obstacles which have so far proved insurmountable in most countries.

The current context of SDGs and UHC contrasts with the Cold War division of worldviews at the time of Alma-Ata,^32^ even as new divisions threaten to escalate. Rather than just affirming primary health care as essential to the SDG and UHC targets, it is useful to discriminate between these two policy vehicles in terms of what each can best guide and achieve. The global health community needs to also consider what else might be required for primary health care to achieve the overlapping visions of health. The discourses around primary health care and UHC intersect and align,^33^ but are not the same.^10^ Primary health care is more strongly linked to primary care and service delivery, while UHC brings an essential and often previously overlooked focus on financing.^12^^,^^34^ Countries’ efforts at implementation of primary health care and UHC demonstrate this. Both primary health care and UHC are only partial paths towards health for all and SDG 3.^35^ We have already noted their deficiencies for tackling the challenges of multisectoral action on the social determinants of health and health security.^26^^,^^36^ The COVID-19 pandemic provides a reminder of the limitations of country capacity, at all income levels, and the supporting global systems to tackle multisectoral health threats and impacts.

At the implementation level, the main determining factors for the success or failure of primary health care lie within countries, notwithstanding the threats to primary health care’s political vision from global challenges such as climate change and the commercial determinants of health. Country needs and actions should be dominant over the preoccupations of global health actors. The specific needs of countries for contextualized knowledge and political strategies should be prioritized over regular efforts to renew global health frameworks. To enhance countries’ chances of success this time, there is a need to be precise and explicit about the scope, tensions, ambiguities and limitations of primary health care, particularly as policy and implementation space. Explanations are needed for how the political vision and implementation focus of primary health care – the idealism of health for all and the pragmatism of primary care – can coexist. Priority for support should be given to low-income and lower-middle-income countries, in particular countries affected by conflict, and to sustained engagement within those countries.

As a contribution to this effort, we present priorities for action for the global system to support country primary health-care efforts (Box 1). These priorities are drawn from our own experience in countries: in supporting primary health care; in attempting to renew primary health care, including during the 30th anniversary of Alma-Ata (and its relative failure); and currently in supporting tackling COVID-19. These proposals are in five areas.

Box 1 **Priority actions for the global system to support countries’ primary health care efforts**

**Assist countries to clarify their priorities for primary health care and monitor implementation, including links to universal health coverage (UHC) and sustainable development goal (SDG) 3^35^**

Support countries to clarify their priority policies and interventions across the three components of the Astana Declaration^4^^,^^5^ in national health plans and strategies.

Update the primary health-care strategy to meet changing and specific country needs.

Plan primary health-care efforts that anticipate countries’ population and health system needs in the future – with robust monitoring of scenarios to allow review and course correction.

Identify and prioritize the needs of countries’ marginalized populations.

Develop indicators for primary health care efforts that go beyond SDG indicators for UHC that reflect only service delivery and financial protection, including indicators for reducing inequities in health service access and outcomes.

**Provide essential technical assistance for primary health care to countries, tailored for different contexts, from fragile states to upper-middle-income and high-income countries**

Provide technical assistance on key areas of the primary health-care strategy, including scale-up and quality improvement of service delivery; health financing mechanisms and defining and costing packages; regulation and governance; digitization; community health systems; and essential public health functions.

Develop and make available high-quality technical tools and strategic guidance that is highly tailored to countries’ individual needs for implementation.

Share countries’ experiences and innovations, and facilitate implementation research.

Pay special attention to the needs of fragile states where primary health-care efforts are most difficult and where the global health community sometimes needs to support service delivery.

**Support countries to navigate the politics of health systems transformation, overcoming resistance to investment and implementation of primary health-care efforts**

Present the specific rationale for the primary health-care strategy that is persuasive to heads of government and ministries of finance.

Redefine the primary care model to improve its appeal to the public, including improving quality and other supply conditions and taking advantage of digitization to build demand and confidence.

Support countries to understand and regulate both for-profit and not-for-profit private sector health services and make strategic choices to steer their contribution to primary health care (and avoid any negative consequences).

**Genuinely align efforts by partners**

Use the *Global action plan for healthy lives and wellbeing for all*^37^ to finally realize partner coordination in countries for primary health care (as envisaged by the inclusion of primary health care as the first accelerator of the plan).

Ensure efforts at partner alignment move beyond consensus and commitments at organizations’ headquarters to tackle common fragmentation of partner interaction on primary health care at the country level.

Accelerate support for the primary health-care strategy from financing institutions, making the health-systems strengthening efforts of vertical programmes a reality.

**Mobilize funding to support primary health-care efforts in countries**

Generate country-specific evidence for investment in the primary health care strategy.

Support ministries of health in negotiations with ministries of finance for funding for primary health care and managing transition of aid to emerging economies.

Provide advice on health financing mechanisms and allocations to support scale-up and to maximize efficiency of the primary health-care strategy.

First, we propose assistance to countries to clarify the specific scope, objectives and priorities for their primary health-care efforts, given the transitions and tensions noted above and the reformulation within the Astana Declaration. This assistance should include considering the model of the future health system to which a country aspires and its intersections and links with UHC and SDG targets, as well as developing monitoring frameworks and building capacity, including ways of reducing health inequities.

Second, there is a need to provide essential technical assistance to countries on primary health care, with a focus on tools and guidance; provide in-country support; share innovations; and build capacity for implementation research. This support needs to be highly customized for different contexts, from fragile states to upper-middle-income and high-income countries. Particular support will be needed for fragile states for whom implementing a primary health-care strategy is both most difficult and has the greatest potential to save lives.

Third, countries need support to navigate the politics of health-systems transformation and to overcome resistance to investment and implementation of primary health-care efforts. The support needs to go beyond health ministries to engagement of heads of government, finance ministries and the public themselves, as well as assistance for stewardship of the private sector.

Fourth, there have been decades of rhetoric on aid, including such concepts as aid harmonization,^38^ diagonal approaches^39^ and positive synergies.^40^ There is now a need for genuine alignment from development partners in supporting national health systems. This support is especially required in the way partners function at country level, away from the compacts and frameworks forged in Geneva, Washington and New York.

Fifth and finally, there remains a major gap in financing for primary health care in most countries, especially in terms of mobilizing domestic resources. This priority is linked to the third area on the politics of primary health care. Countries need support in understanding what needs to be financed to meet people’s expectations of health care, in making the case for investment and the use of mechanisms such as consumption taxes,^41^ and in increasing efficiency.

## Primary health care and COVID-19

The response to the COVID-19 pandemic shows the missed opportunities for advancing health for all through primary health care. In countries of all income levels, with a few notable exceptions, the role of primary care in COVID-19 – and the application of primary health care as a strategy – has been limited. Primary care facilities have been bypassed for coordinating and conducting specimen collection for testing. Community cadres have been underused for surveillance and community engagement. Clinical care has focused on hospitals, with greater roles for hospitals in patient care facilitated by telemedicine. Restrictions on people’s movements have resulted in lower health service utilization and even threatened the financial viability of some primary care facilities. Support from development partners calling for and guiding a primary health-care strategy to COVID-19 has been lagging.

It is understandable in a crisis of this magnitude that attention and resources go to health-systems responses that are most feasible and seem to be the most immediately effective. But the COVID-19 response is an indictment of the failures of the primary health-care movement. The response demonstrates that health systems in most countries are not sufficiently oriented towards primary health care to be mobilized in a crisis. The lessons of the Ebola virus disease outbreak 2013–2016, when discussions highlighted the importance of community leadership and resilient health systems,^42^ have not been learnt. Primary care services and CHW cadres in most countries still lack the capacity or the policy environment to be the fulcrum of the COVID-19 health service response. Primary care is thus not enabled to contribute substantively to outbreak surveillance and response or to undertake community-based care with sufficient confidence in infection prevention and control and effective referral mechanisms. All of us who claim to be primary health-care proponents should reflect on these failures and our own accountability. Capacity-building will require that initiatives to build global health security are not an isolated activity, but genuinely linked to efforts at health-systems strengthening. In this respect, there are worrying signs of further parallel initiatives, as often occurs in response to crises.^43^^,^^44^

## Conclusion

The Declaration of Alma-Ata and primary health care remain rightly celebrated, despite decades of unrealized potential and the tension between the political vision of health for all and the implementation vehicle of primary care. Clarifying this tension is one contribution to rewriting upon the palimpsest of the Alma-Ata Declaration to accommodate changing contexts and realities. A clear distinction is needed between primary health care as an inspirational vision and set of values for health development, and primary health care as policy and implementation space. To achieve this vision, political action is required. Stakeholders beyond the health sector will often need to lead, which is challenging because the concept of primary health care is poorly understood by other sectors.

Efforts on primary health care as policy and implementation space might focus on the key directions we have described above in this paper, as partial but important implementation solutions to contribute to the much bigger political vision of primary health care. These directions entail an explicit focus on primary care and the frontline of service delivery with clear links and support to complementary work on social determinants and building healthy societies. The heterogeneity of contexts for primary care in the modern world need to be recognized and addressed. Social and technological innovations can be better harnessed and applied widely, including in building capacity in primary care and community systems to tackle health security challenges revealed by COVID-19. All the diverse providers that deliver primary care require engagement and stewardship, understanding the links between them as part of national health systems, to maximize their contribution to health for all. 
